# Apaf-1 Inhibitors Protect from Unwanted Cell Death in *In Vivo* Models of Kidney Ischemia and Chemotherapy Induced Ototoxicity

**DOI:** 10.1371/journal.pone.0110979

**Published:** 2014-10-20

**Authors:** Mar Orzáez, Mónica Sancho, Sandra Marchán, Laura Mondragón, Rebeca Montava, Juan García Valero, Olatz Landeta, Gorka Basañez, Rodrigo J. Carbajo, Antonio Pineda-Lucena, Jordi Bujons, Alejandra Moure, Angel Messeguer, Carmen Lagunas, Carmen Herrero, Enrique Pérez-Payá

**Affiliations:** 1 Laboratory of Peptide and Protein Chemistry, Centro de Investigación Príncipe Felipe, Valencia, Spain; 2 Laboratorios SALVAT S.A., Esplugues de Llobregat, Barcelona, Spain; 3 Unidad de Biofísica, CSIC, UPV/EHU, Leioa, Spain; 4 Laboratory of Structural Biochemistry, Centro de Investigación Príncipe Felipe, Valencia, Spain; 5 Department of Chemical and Biomolecular Nanotechnology and Department of Biological Chemistry and Molecular Modeling, Instituto de Química Avanzada de Cataluña (CSIC), Barcelona, Spain; 6 Instituto de Biomedicina de Valencia (CSIC), Valencia, Spain; Karolinska Institutet, Sweden

## Abstract

**Background:**

Excessive apoptosis induces unwanted cell death and promotes pathological conditions. Drug discovery efforts aimed at decreasing apoptotic damage initially targeted the inhibition of effector caspases. Although such inhibitors were effective, safety problems led to slow pharmacological development. Therefore, apoptosis inhibition is still considered an unmet medical need.

**Methodology and Principal Findings:**

The interaction between Apaf-1 and the inhibitors was confirmed by NMR. Target specificity was evaluated in cellular models by siRNa based approaches. Cell recovery was confirmed by MTT, clonogenicity and flow cytometry assays. The efficiency of the compounds as antiapoptotic agents was tested in cellular and *in* *vivo* models of protection upon cisplatin induced ototoxicity in a zebrafish model and from hypoxia and reperfusion kidney damage in a rat model of hot ischemia.

**Conclusions:**

Apaf-1 inhibitors decreased Cyt*c* release and apoptosome-mediated activation of procaspase-9 preventing cell and tissue damage in *ex* *vivo* experiments and *in* *vivo* animal models of apoptotic damage. Our results provide evidence that Apaf-1 pharmacological inhibition has therapeutic potential for the treatment of apoptosis-related diseases.

## Introduction

The intrinsic or mitochondria mediated apoptosis pathway can be initiated by a number of cellular stress factors that together with the participation of members of the BCL-2 family of proteins, lead to mitochondrial outer membrane permeabilization (MOMP) [1]. This is followed by cytochrome *c* (Cyt*c*) release from mitochondria that binds to the protein Apaf-1 (apoptotic protease-activating factor) [2] and forms the multiprotein complex termed apoptosome. The apoptosome recruits and activates an initiator member of the caspase family of cysteine aspartyl proteases, procaspase-9, that in turn activates apoptosis-effector caspases initiating therefore apoptotic cell death. Defects in the regulation of apoptosis are at the root of a variety of diseases. When cells acquire resistance to apoptosis it frequently correlates with cancer or autoimmune diseases. In contrast, excessive apoptosis induces unwanted cell death and promotes pathological conditions related to stroke, ischemia-reperfusion damage and degenerative diseases [3]. Therefore, there is a medical need for treatments based on unwanted apoptosis inhibition, but no treatment has been approved. In this sense, drug discovery efforts initially targeted the inhibition of caspase activity, particularly the executioner caspase-3 [4]. This strategy demonstrated a promising potential in several animal models [5], [6], [7], [8] although caspase inhibitor-based drugs are experimenting slow pharmacological advance due to the reported side effects [9].

To address the goal of developing unwanted apoptosis inhibitors new pharmacological targets of the apoptotic pathway have to be explored. Of special interest are the protein-protein interactions upstream of caspase activation in particular, the formation of the apoptosome offered evidences to be considered as an interesting target for developing anti-apoptotic therapies [10], [11]. The main constituent of the apoptosome is Apaf-1, a protein involved in nucleotide and Cyt*c* binding [12]. Apaf-1 is a multidomain protein with an N-terminal caspase recruitment domain (CARD), a central nucleotide-binding and oligomerization domain (NOD), and a C-terminal WD40 repeats domain. We previously reported on a first generation of small molecules that inhibit apoptosis by interfering with the apoptosome activity [13], [14]. In particular, SVT016426 (previously named QM31 [14] is an efficient inhibitor of apoptosis. Here we demonstrated that SVT016426 specifically targets Apaf-1 inhibiting the activation of procaspase-9 *in* *vitro* and *in cellulo*-based systems. The treatment of apoptosis injured cells with SVT016426 and its derivatives showed a decrease in Cyt*c* release from mitochondria and an improvement in cell viability. We provide evidences that a single target could define a pharmacological alternative that prevents mitochondrial damage and caspase activation and present proof of principle for therapeutic relevance in inhibition of unwanted apoptosis in animal models.

## Results

### Apaf-1 inhibitors

**Figure 1:**
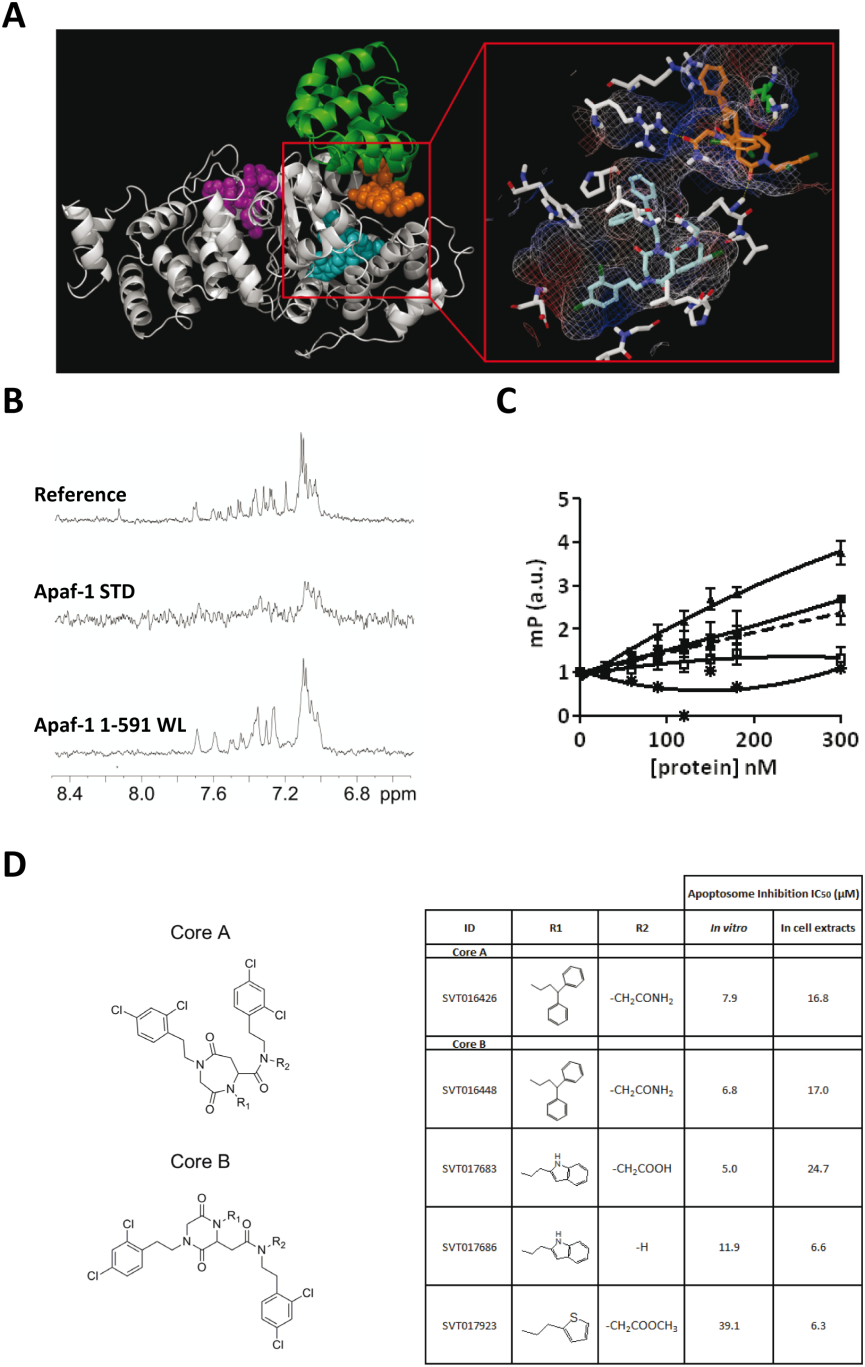
Design of new Apaf-1 inhibitor derivatives (SVTs). (**A**) Human Apaf-1 1–591 structure showing the CARD (green) and NOD (light gray) domains, as well as the best docked poses, for SVT016426 at the CARD-NOD interface (orange) and at the nucleotide binding site (cyan). The inset shows also the interacting Apaf-1 residues (see File S1). (**B**) Details of the aromatic region of NMR spectra acquired with Apaf-1 and Apaf-1 1–591 in the presence of SVT016426. Under the experimental conditions, only the signals from SVT016426 are observed. From top, one dimension ^1^H NMR reference spectrum of SVT016426; Saturation Transfer Difference experiment (STD) and WaterLOGSY for SVT016426 with Apaf-1 and Apaf-1 1–591. (**C**) CF-SVT016426 fluorescence polarization assays for Apaf-1 and Apaf-1 1–591 in the presence or absence of dATP (100 µM) (n = 3). (**D**) The IC_50_s of SVTs were analyzed using *in* *vitro* reconstitution of the apoptosome and HEK293 cell extract-based assays (see Materials and Methods and Fig. S3A and S3B in File S1).

SVT016426 was discovered as a result of an initial medicinal chemistry program directed to improve a series of linear peptidomimetics discovered as inhibitors of the activity of the apoptosome *via* chemical library screening [13]. The compound inhibits anthracyclin-induced apoptosis in a variety of transformed human cell lines and cell death induced by doxycycline-inducible BAX overexpression in human osteosarcoma cells [13]. These results suggest that SVT016426 may constitute a new class of cytoprotective agents. One of the main concerns to achieve *in* *vivo* experiments with the SVT016426 was the low solubility of this drug. Then, we initiated a study of the putative binding site of the compound on the surface of Apaf-1 to obtain information for the design of SVT016426-derivatives. A blind docking screening targeting the reported human WD40 repeats depleted Apaf-1 (Apaf-1 1–591) structure [15] revealed potential binding sites for SVT016426 at the CARD-NOD interface and at the reported dATP binding site in the NOD domain [15] (Fig. 1A; Table S1 and Fig. S1 and S2 in File S1). Thus, binding of SVT016426 could either stabilize Apaf-1 into a “locked” conformation, which may hinder unpacking of the CARD-NOD interface that facilitates nucleotide binding or directly block the nucleotide binding site. Furthermore, we confirmed by NMR-based experiments that there was binding to Apaf-1 and Apaf-1 1–591 (Fig. 1B). We applied two complementary ligand-based NMR techniques that analyze the effects of ligand binding on NMR signals: waterLOGSY (water-ligand observed by gradient spectroscopy) [16] and STD (saturation transfer difference) [17]. In these techniques, an excess of ligand is mixed with the target protein (here, SVT016426 and Apaf-1), and the exchange between the bound and free states of the ligand modulates the NMR signal of the free ligand. Both STD and waterLOGSY, experiments produced positive interaction results with Apaf-1 and Apaf-1 1–591 constructs (Fig. 1B). In addition, we used a carboxyfluorescein-labelled derivative of SVT016426 (CF-SVT016426) and fluorescence polarization spectroscopy to demonstrate that SVT016426 bound to recombinant Apaf-1 and to recombinant Apaf-1 1–591 (Fig. 1C). dATP decreased the affinity, suggesting that the Apaf-1 binding site for SVT016426 involves the CARD and NOD domains. Based on the structural information we generated a number of SVT016426-derivatives in a medicinal chemistry effort focused on identifying compounds with similar activities but better pharmacological properties that were amenable to the different therapeutic applications. Then, we synthesized compounds with a six-member ring in the central core of the molecule (core B Fig. 1D) and different substituents, generating the compounds listed in Fig. 1D. All of them inhibited the activation of procaspase-9 in an *in* *vitro* reconstituted apoptosome [18] and in a cell extract-based assay [13] (Fig. 1D; Fig. S3A and S3B in File S1). To confirm apoptosome inhibiting activity, the caspase-9 processing was also followed by immunoblotting in cellular assays (Fig. S4 in File S1). However, SVT compounds did not have a direct inhibitory effect on recombinant caspase-3 and caspase-9 and did not show features of being a promiscuous aggregator in the β-lactamase inhibition assay [19] (Fig. S3C–S3E in File S1).

### The protective effect of SVT016426 requires the presence of Apaf-1 in the cell

**Figure 2:**
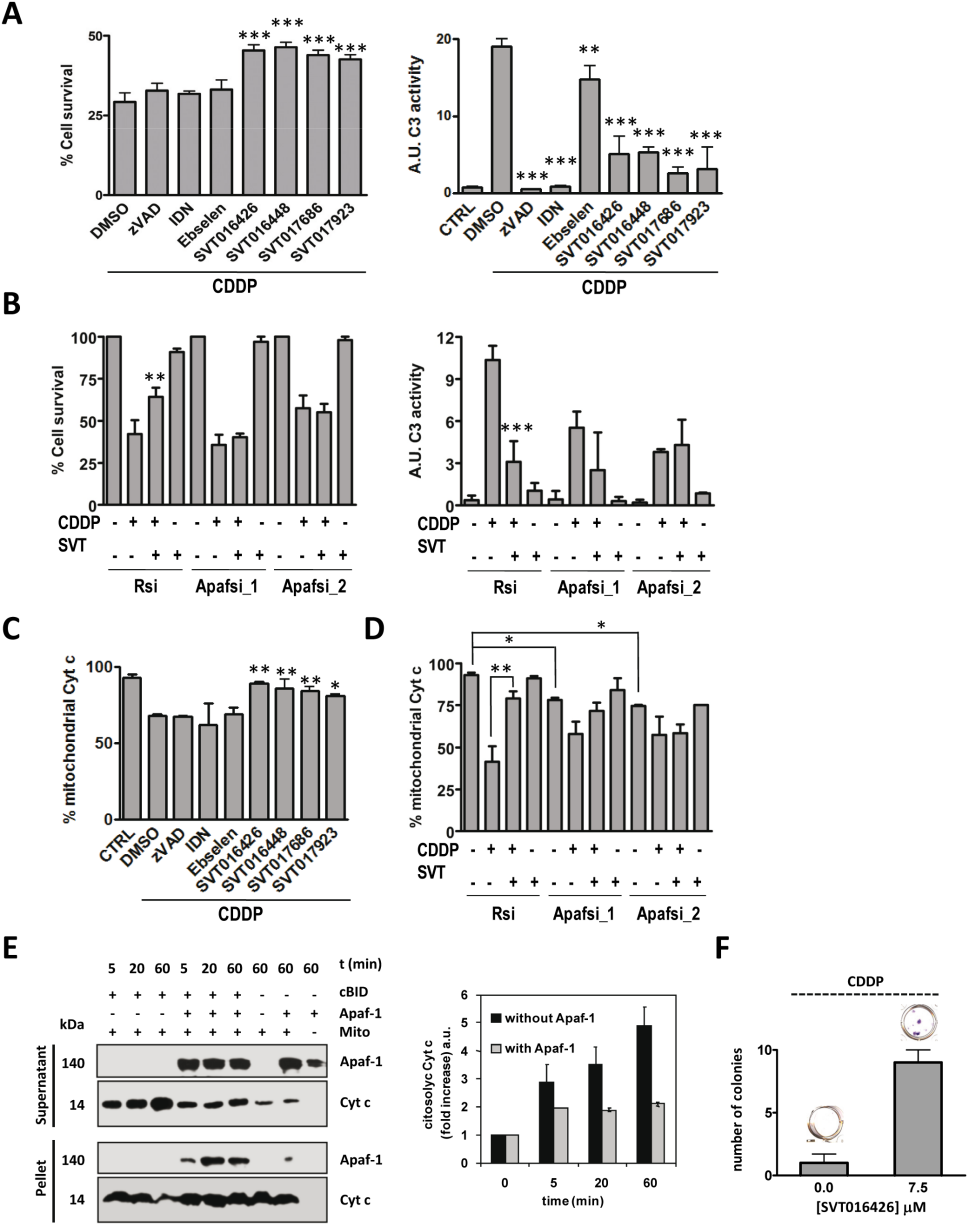
Apaf-1 is required for the inhibitory activity of SVT016426. (**A**) From left to right, cell survival and caspase-3 activity of wtMEFS after 30 h incubation with CDDP (20 µM) in the presence of vehicle (DMSO), zVAD (1 µM), IDN6566 (IDN; 1 µM), Ebselen (10 µM), or SVT016426-derivatives (10 µM) (mean ± SD, n = 3 independent experiments). (**B**) Apaf-1 was silenced in HeLa cells by two different Apaf-1 siRNAs (100 nM). Cell survival and caspase-3 activity were measured for control (Rsi) or Apaf-1 (Apafsi) silencing in the presence or absence of SVT016426 treatment (SVT; 10 µM) (mean ± SD, n = 3 independent experiments). Asterisks represent significant differences as determined by one-way ANOVA test with Bonferroni’s multiple comparison post-test (*p<0.1; **p<0.05; ***p<0.001). (**C–D**) Analysis of mitochondrial Cyt*c* by cytometry analysis in the A and B described conditions. (**E**) Apaf-1 binds to mitochondria in a cBID dependent manner and inhibits Cyt*c* release in purified mitochondria. The mitochondria treated with rApaf-1 (150 nM) and cBID (10 nM) were centrifuged and separated into pellet (mitochondria) and supernatant and immunobloted for Cyt*c* release and Apaf-1 incorporation (left panel). Bands were quantified by densitometry (right panel). (**F**) wtMEFS were treated with 5 µM CDDP for 6 h and incubated in the presence or absence SVT016426. After two weeks cells were fixed and stained with crystal violet and the quantification of the number of cell colonies was performed. Data represent the mean ± SD of three independent experiments.

Excessive apoptosis is a problem associated with many diseases and organ-stress processes that remains to be solved from the pharmacological point of view. Current anti-apoptotic drugs such as caspase inhibitors seem to act too late in the cell death process and recovery results are being not as good as expected. Then, in order to rationalize the advantages of working with Apaf-1 inhibitors, we performed a comparative study of the Apaf-1 inhibition-cell recovery potential with other apoptosis inhibitors in wild type mouse embryonic fibroblasts (wtMEFS) (Fig. 2A) and HeLa cells (Fig. S5A in File S1). We have included in this study caspase-3 inhibitors, such as z-VAD-fmk and IDN-6556 [20], and the antioxidant ebselen. Ebselen (2-phenyl-1,2-benzisoselenazol-3[2H]-one) is a selenoorganic compound exhibiting both glutathione peroxidase activity and antioxidant activity which has been reported to inhibit apoptosis in several models [21]). We used a cell-based model to demonstrate that after treatment with the intrinsic apoptotic inducer cisplatin (cis-diammineplatinum(II) dichloride, CDDP), cell death in wtMEFS was inhibited by SVT016426 and its derivatives (Fig. 2A and Fig. S5A in File S1). While all the compounds decreased CDDP-induced caspase-3 activity; only Apaf-1 inhibitors diminished Cyt*c* release and cell death, improving survival (Fig. 2A and 2C; Fig. S5A in File S1).

In order to show that all the observed effects were dependent on Apaf-1 inhibition, we demonstrated that CDDP-induced cell death was not inhibited by SVT016426 in Apaf-1 siRNA-based knockdown HeLa cells; although, it did inhibit CDDP-induced cell death in control cells transfected with random siRNA (Fig. 2B and Fig. S5B in File S1). These results correlated with measurements of caspase-3 activity and Cyt*c* release from mitochondria (Fig. 2B and 2D), suggesting that the inhibitory capacity of SVT016426 was dependent on the levels of Apaf-1 in the cell. Equivalent results were observed with the different SVT016426-derivatives (Fig. S5C in File S1). We also evaluated several intrinsic apoptosis inducers, such as etoposide, with similar results (data not shown). Taken together, these results confirmed that SVT016426 and its derivatives avoid apoptosis activation through an inhibitory effect on Apaf-1 activity and that the cytoprotective effect of the compounds requires the presence of Apaf-1 in the cell.

Remarkably, silencing of Apaf-1 renders cells with less mitochondrial Cyt*c* in the two different Apaf-1 siRNA assayed (Fig. 2D) suggesting a role for Apaf-1 in the dynamics of Cyt*c* release. Supporting this hypothesis, the addition of rApaf-1 to isolated mitochondria inhibits cBid mediated Cyt*c* release (Fig. 2E). The levels of mitochondrial Cyt*c* in the presence of the apoptotic inductor CDDP decrease, but are recovered when cells are treated with SVT016426 (Fig. 2D). This effect is lost when Apaf-1 is silenced, ruling out the existence of an SVT016426 off-target responsible of this effect. Then we propose a SVT016426 mechanism of action where the inhibition of the apoptosome function of Apaf-1 promotes its interaction with the mitochondria avoiding Cyt*c* release, and thus improving cell survival. In fact, treatment with CDDP in the presence of SVT016426 recovers mitochondrial membrane permeability (Fig. S6 in File S1) and allows partial clonogenic recovery on MEFS cells (Fig. 2F). These results open the field to future studies about a putative role of Apaf-1 in mitochondrial function.

### Apaf-1 inhibitors prevent unwanted apoptosis in animal models of disease

**Figure 3:**
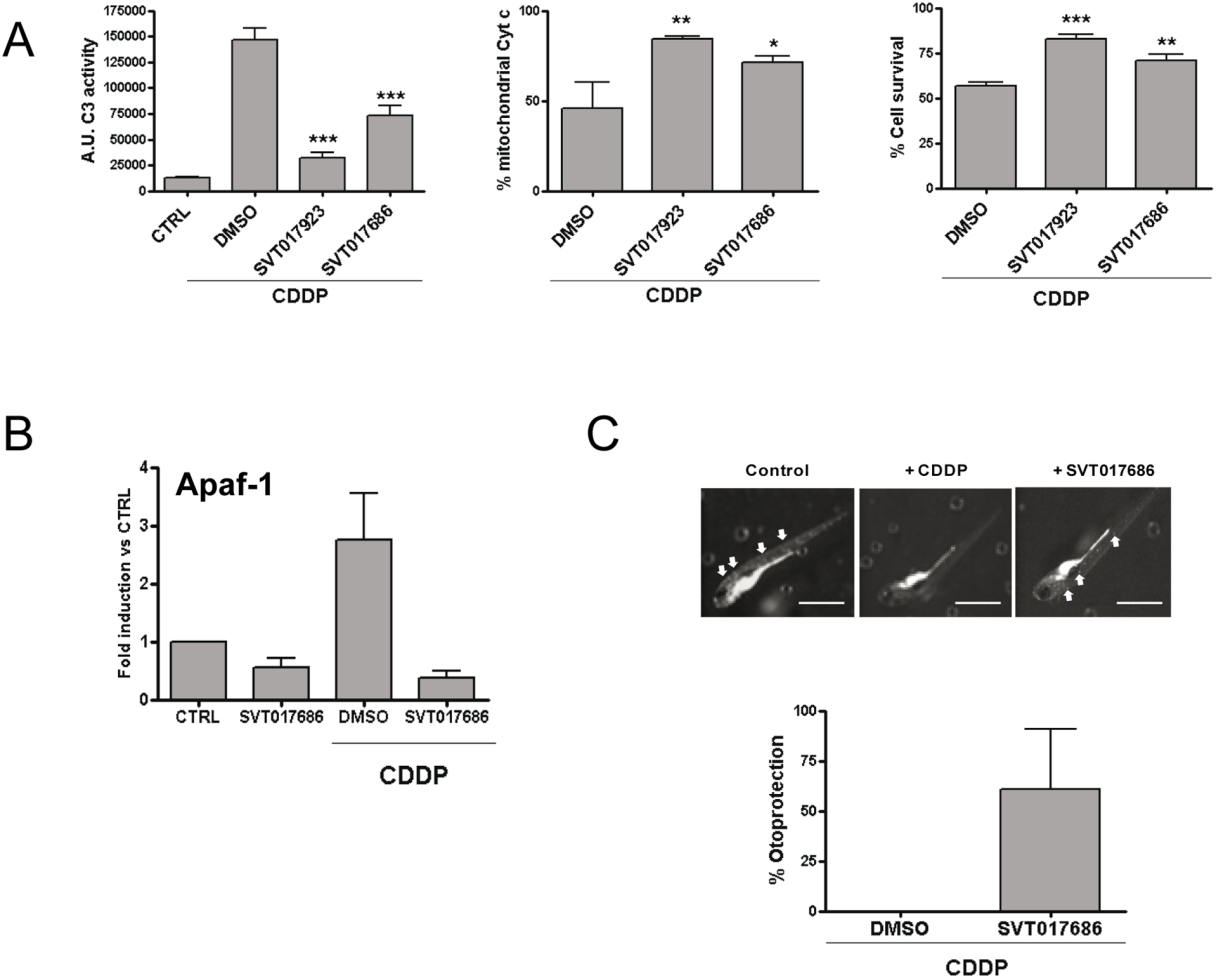
Apaf-1 inhibitors protected against CDDP-induced ototoxicity in an *in* *vitro* and *in* *vivo* model. (**A**) Caspase-3 activity, Cyt*c* release and cell survival, respectively from HEI-OC1 cells treated with CDDP (25 µM) in the presence or absence of compounds (10 µM). Data are expressed as the mean ± SD (n = 3 independent assays). Asterisks represent significant differences relative to DMSO treatment as determined by one-way ANOVA test with Bonferroni’s multiple comparison post-test (*p<0.1; **p<0.05; ***p<0.001). (**B**) Transcriptional regulation of Apaf-1 analyzed by RT-qPCR from HEI-OC1 cells treated with CDDP (25 µM) in the presence or absence of compounds (10 µM) (mean ± SD; n = 3). (**C**) Apaf-1 inhibitors protected against CDDP-induced ototoxicity in a zebrafish model. Five-day embryos (n = 10) were treated with CDDP (10 µM) for 24 h in the presence or absence of SVT017686 (15 µM). Neuroblasts are the white dots (arrows). Scale bar, 1 mm. Hair cells were counted and cell survival was calculated as a percentage of the control group (not exposed to CDDP). Data bars represent mean ± SD hair cell survival (n = 9/10 fishes).

CDDP-based chemotherapy has wide application to treat different cancers although not devoid of side effects. CDDP induced ototoxicity is one of the main dose-limiting side-effect of anti-neoplastic treatment. In CDDP-therapy, the drug accumulates in the inner ear fluids and is then taken up by otic epithelial cells, particularly in the cochlea [22] inducing ototoxicity that can lead to permanent hearing loss. At the molecular level, CDDP triggers the reactive oxygen and nitrogen species production inducing thereby apoptosis cell death of inner hair cells. The efficacy of Apaf-1 inhibitors, SVT017686 and SVT017923, in preventing CDDP-induced apoptosis was tested in an organ of Corti-derived cell line (HEI-OC1). CDDP treatment induced apoptotic cell death characterized by caspase-3 activation and Cyt*c* release. Both were decreased in the presence of the compounds and this effect was translated in an improved cell survival (Fig. 3A). Compound treatment reduced the expression of Apaf-1 which was increased after CDDP treatment (Fig. 3B). These results suggest that Apaf-1 inhibition has a cytoprotective effect in HEI-OC1 cell line after CDDP-induced cell death.

Finally, in order to probe the *in* *vivo* efficacy of SVT017686, a zebrafish model of CDDP induced ototoxicity was set up [23]. Fig. 3C shows the distribution of auditory neuromasts in 5 dpf zebrafish, as detected by DASPEI staining. SVT017686 markedly decreases the CDDP induced loss of neuromast, demonstrating that Apaf-1 inhibition is an effective strategy against CDDP induced ototoxicity.

**Figure 4:**
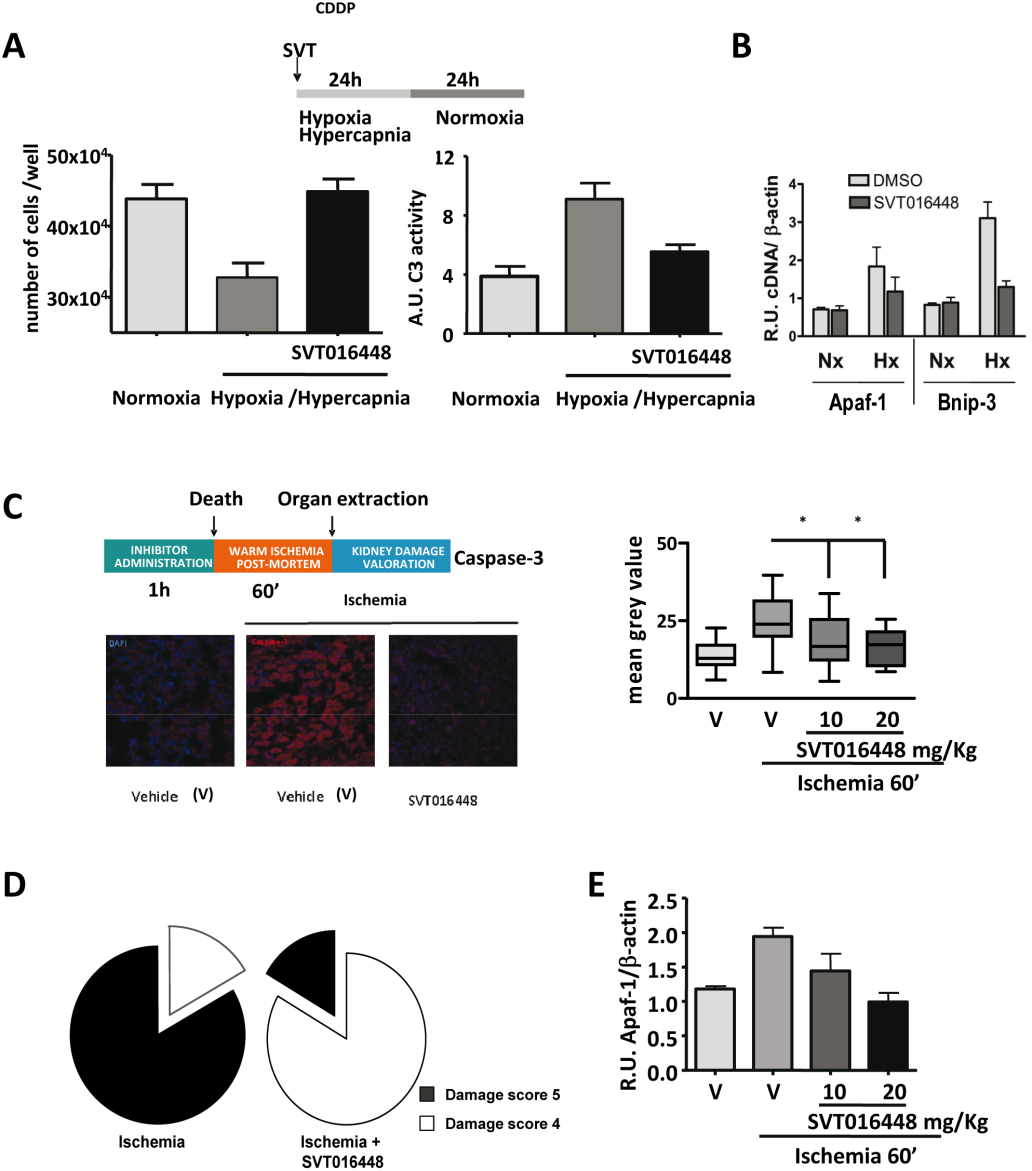
Apaf-1 inhibitors provide protection in an *in* *vivo* animal ischemic induced acute kidney injury model. (**A**) Cell survival and caspase-3 activity in cell lysates from LLC-PK-1 cells measured after 24 h hypoxia/hypercapnia plus 24 h normoxia in the absence or presence of Apaf-1 inhibitor (10 µM) (mean ± SD; n = 3). (**B**) Transcriptional regulation of Apaf-1 and Bnip-3 were analyzed by RT-qPCR in LLC-PK-1 cells. mRNA levels were compared in hypoxia/hypercapnia (Hx) *versus* normoxia (Nx) conditions after SVT016448 treatment (mean ± SD; n = 3). (**C**) Intraperitoneal injection of SVT016448 (10 mg/Kg) decreases caspase activity in a renal hot ischemia *in* *vivo* model. A representative image of the immunofluorescence of active caspase-3 (left panel) and mean grey value quantification (right panel) (mean ± SD, n = 5). Asterisks represent significant differences relative to ischemia treatment as determined by one-way ANOVA test with Bonferroni’s multiple comparison post-test (*p<0.05). (**D**) Damaged tissue decreases in the presence of the Apaf-1 inhibitor, as determined by histopathological evaluation. (**E**) SVT016448 treatment-dependent decrease in Apaf-1 mRNA levels, as analyzed by RT-qPCR.

Ischemia and reperfusion (I/R) is a pathological condition characterized by blood restriction-dependent tissue hypoxia. In renal transplantation, I/R is associated with pathophysiological alterations (tubular epithelial cells undergo necrosis and apoptosis) resulting in the destruction of renal tissue and delayed graft function, affecting the long-term transplant outcome [24], [25]. We evaluated whether the beneficial effects observed for SVT016426 and its derivatives on chemotherapeutic-induced cell death could be applied to hypoxia-induced apoptosis. We used cultured porcine proximal renal tubule (LLC-PK-1) cells treated with SVT016448 (this compound was more active than SVT016426 in the experimental setup procedures with these cells). The cells were exposed to hypoxia/hypercapnia (HH) conditions for 24 h and then to normoxia for 24 h to simulate I/R. Apoptosis was reduced in SVT016448-treated cells, as measured by increased cell viability and a decreased level of I/R-induced caspase-3 activity (Fig. 4A). The HH treatment led to transcriptional upregulation of Apaf-1 at the same levels observed for Bnip3 mRNA after hypoxia-inducible factor 1α (HIF-1α) pathway activation [26] (Fig. 4B), and both levels were inhibited by SVT016448 treatment. If functional problems derived from hot ischemia injury could be overcome, kidneys from non-heart-beating donors could be used for transplantation, which would increase the number of organs available. To determine the impact of Apaf-1 inhibition *in* *vivo*, we evaluated SVT016448 in a rat model of hot ischemia [27]. Immunofluorescence labeling of kidney slices for active caspase-3 showed that animals treated with intraperitoneal injections of SVT016448 had lowered levels of apoptosis (Fig. 4C) and less kidney lesions in blind histopathological analysis (Fig. 4D). RT-qPCR analysis of kidney tissue showed that SVT016448 treatment lowered the expression of Apaf-1, which was overexpressed after hot ischemia treatment (Fig. 4E). Similar results were found when the compound was supplied intravenously (Fig. S7 in File S1). Thus, Apaf-1 can be defined as pharmacological target for inhibition of unwanted apoptosis.

## Discussion

Apoptosis is on the basis of numerous pathologies but there is not an approved treatment based on its inhibition. Previous proposals on apoptosis inhibitors faced with troubles in development. Here, we describe a new pharmacological alternative with two fold significance. First, we characterized SVT016426 and its derivatives as selective inhibitors of Apaf-1, which lead to decreased unwanted cell death in animal models of excessive apoptosis-related pathological disorders. Second, the results obtained when apoptotic injured cells were treated with SVT016426 suggested a mitochondrio-protective effect that prompted to seek for a role of Apaf-1 at Cyt*c* release from mitochondria.

We propose that the SVT family of Apaf-1 inhibitors binds to Apaf-1 at the CARD-NOD interface or at the reported dATP binding site in the NOD domain and thwarted the required conformational change that permits a productive Apaf-1 oligomerization [28]. As a consequence the recruitment of procaspase-9 is not effective, inhibiting the induction of the apoptotic pathway in cells [13]. The SVT family has not unspecific cell toxicity and did not show any noticeable effect in cells where Apaf-1 was not present by gene knock down by siRNA (Fig. 2). In addition we found that long term treatment with Apaf-1 inhibitors did not induce alterations in cell proliferation and aneuploidy (Fig. S8 in File S1). Caspases have been considered as the effectors of cell death during apoptosis then caspase inhibitors were therapeutically evaluated in apoptosis related diseases [4], [7]. Furthermore, due to the MOMP-dependent generation of reactive oxygen species, different antioxidants as ebselen or free radical scavengers as N-Acetylcysteine (NAC) have been also proposed as agents to palliate the noxious consequences of unwanted apoptosis [21], [29]. There were problems determining the clinical benefit of NAC [30] and although the pan-caspase inhibitor IDN-6556 was effective in different settings [6], [7], [8] safety concerns were raised due to atypical cellular infiltrate in the liver, kidney, and adrenal glands in experimental models [9]. Here we demonstrated that ebselen, IDN-6556 as well as the SVT compounds, showed inhibition of caspase activity (Fig. 2B) however only SVT compounds showed potential to prevent CDDP-, and hypoxia-induced cell death (Fig. 2 and 4). In fact, in mammals solely caspase inhibition, downstream of MOMP, delays and modifies the outcome, more than prevents cell death [31]. Strikingly, in all the cell types analyzed in our study the SVT-dependent cell death inhibition correlated with a decrease in Cyt*c* release from mitochondria. In contrast, no such effect was observed in cells treated with caspase inhibitors or antioxidants. We conclude that SVT-compounds were inducing a pre-MOMP event probably related to Apaf-1. Previous reports include conflicting data on the role of Apaf-1 on Cyt*c* release. Franklin and Robertson [32] showed that in Apaf-1 deficient Jurkat T-lymphocytes etoposide- and mitoxantrone-induced Cyt*c* release and loss of mitochondrial membrane potential was impaired. In contrast, Ferraro *et al*. [33] observed that Cyt*c* release from Apaf-1-deficient proneural embryonic telencephalic naïve *Apaf-1* knockout cells, followed faster kinetics than that of wild type cells. In addition, Potokar *et al*. [34] observed that at short times after rotenone-induced apoptosis in pituitary cells Apaf-1 redistributed to mitochondria. Our *in* *vitro* experiments with isolated mitochondria aiming to reproduce the initial events of mitochondria-mediated apoptosis using cBID showed [35] that the presence of Apaf-1 on mitochondria cBID-induced attenuated release of Cyt*c* (Fig. 2) and thus Apaf-1 is probably a modulator of MOMP events. The Cyt*c* release inhibitory capability of SVT016426 was dependent on the Apaf-1 expression levels in the cell. Moreover, Apaf-1 gene silencing renders cells with less mitochondrial Cyt*c* (Fig. 2D). All together, our results shift the balance to the existence of a role for Apaf-1 in the control of Cyt*c* release.

Nowadays there is no ideal protective agent in clinical use against cisplatin ototoxicity. This would eliminate one of the dose-limiting side effects of cisplatin therapy and improve the quality of life for many patients [36]. As an example clinical trials for the transtympanicof L-N-Acetylcysteine administration in patients receiving cisplatin chemotherapy for head and neck cancerdid not demonstrate a significant benefit [37]. There is a great need to find safe and effective protective agents. In this study we show the effectiveness of treatment with SVTs in cellular and in vivo models of cisplatin- induced ototoxicity.

Ischemic, toxic, or obstructive renal damage triggers apoptotic mechanisms that cause tubular cell loss and decreased renal function [38]. Currently there are no effective pharmacological strategies to treat acute renal failure. Here, we show the ability of Apaf-1 inhibitors to decrease cell death in kidney ischemia cellular and *in* *vivo* models. These results support the potential application of Apaf-1 inhibitors to prevent acute kidney injury (AKI).

In conclusion, we characterized SVT016426 and its derivatives as selective inhibitors of Apaf-1 that prevent cell death in different cellular settings and show efficiency as potential apoptosis inhibitory drugs in models of disease.

## Materials and Methods

### Chemical synthesis

The general method for the synthesis of perhydro-1,4-diazepine-2,5-diones such as SVT016426 and 3-substituted 1,4-piperazine-2,5-dione derivatives (SVT016448, SVT017686 and SVT017923) has been previously reported [39].

### Apoptosome activity assays

Apoptosome was reconstituted by incubating rApaf-1 (100 nM) with vehicle (DMSO) or SVT compounds in assay buffer (20 mM HEPES, 10 mMKCl, 1.5 mM MgCl_2_, 1 mM EDTA, 1 mM EGTA, 1 mM DTT, 0.1 mM PMSF) at 30°C for 30 min. Next, 100 µM dATP and 100 nM Cyt*c* were added and incubated for additional 40 min. Then, caspase-9 (100 nM) was added, and activity was monitored by the Ac-LEHD-afc substrate (50 µM; Enzo Life Sciences) using a Victor 2 spectrofluorimeter (λ_exc_ = 390 nm; λ_em_ = 510 nm). A cell-free caspase activation assay was also performed as described [13], [40]. Briefly, Apaf-1 depleted cytosolic extracts from HEK293 were incubated with rApaf-1 (100 nM) and in the presence of the different SVT-derivatives. Ac-DEVD-afc substrate (Enzo Life Sciences) was used to measure caspase 3 activity, using a Victor 2 spectrofluorimeter.

### Fluorescence polarization spectroscopy

A 60 nM solution of 5′-6′carboxyfluorescein-labeled SVT016426 was titrated with concentrated protein solutions in reaction buffer (total reaction volume was 200 µl) Fluorescence polarization measurements were recorded in a Victor 2 Wallac 1420 Workstation spectrofluorimeter (λexc = 480 nm; λem = 535 nm).

### Computational docking

The blind docking protocol consisted on a search for cavities in the Apaf-1 CARD-NOD structure to identify potential ligand binding sites, followed by a flexible docking of the ligand on to the different sites located. Fig. S1 in File S1 shows the best scored sites for interaction within the target structure and Table S1 in File S1 summarizes the site scores. Docking at the binding sites rendered plausible binding poses for compound SVT016426 (Fig. S2 and Table S1 in File S1).

#### Computational Methods

Molecular simulations were conducted with the package Schrödinger Suite 2011 [41] through its graphical interface Maestro [42]. The program Macromodel [43], with its default force field OPLS 2005, a modified version of the OPLS-AA force field [44], and GB/SA water solvation conditions [45] were used for all energetic calculations. The coordinates of WD40-deleted human Apaf-1 (Apaf-1 1–591, PDB 1Z6T, chain B) were obtained from the Protein Data Bank [46] at Brookhaven National Laboratory. The structure of the protein was prepared using the Protein Preparation Wizard included in Maestro to remove the unused subunits as well as the solvent molecules and ligands, adding hydrogens, setting protonation states and minimizing the energy using the OPLS force field. SiteMap [47], [48], [49], was used to identify and score potential binding sites on the protein. The score provided by SiteMap is constructed and calibrated so that the average SiteScore for 157 investigated submicromolar sites is 1.0. Therefore, a score≥1.0 suggests a site of particular promise, while a score≤0.8 has been found to accurately distinguish between drug-binding and non-drug-binding sites. The best scored sites were used as targets for docking of the SVT016426 ligand with the program Glide XP [50], [51], [52]. The structure of SVT016426 was built within Maestro and then it was prepared with the LigPrep application [53] included in the software to generate ring conformers of the two stereoisomers of the compound. Eight ring conformers for each enantiomer were generated and used as input ligands for the docking at each target site. In order to ensure a good conformational sampling during the docking simulations the following settings were used: a maximum of 5000000 poses per input structure for the initial phase of docking, the extendend sampling protocol, and 50 poses for the post-docking minimization. Glide XP scores were used to rank the resulting docked poses. The interaction diagrams for the best poses were built with the Ligand Interaction Diagram application implemented in Maestro.

### NMR experiments

NMR spectra for protein ligand interaction were recorded at 298 K with a BrukerUltrashield Plus Avance II 600 MHz spectrometer equipped with a 5-mm cryogenically-cooled TCI probe. An NMR sample consisted of 1 µM rApaf-1 or Apaf-1 1–591 and 20 or 100 µM SVT017683 to yield protein:ligand ratios of 1∶20 and 1∶100 for the waterLOGSY and STD experiments, respectively. The sample buffer was 50 mM phosphate, pH 7.0, with 50 mMNaCl; 8 K points were used for a sweep width of 9,600 Hz and a total of 1 K and 4 K scans were accumulated for the waterLOGSY and STD experiments, respectively.

### Cell culture and reagents

Human cervix adenocarcinoma (HeLa) and proximal tubular renal porcine LLC-PK-1 cells were obtained from ATCC (Rockville, MD). wtMEFS, previously established in the referenced publications [54], were kindly provided by Dr. Franceso Cecconi. The HEI-OC1 cell line (House Ear Institute-Organ of Corti-1) was obtained from F. Kalinec and primary human dermal fibroblasts (HDFn) were obtained from Cascade Biologics. HeLa, HDFn, wtMEFS cells were cultured in DMEM supplemented with 10% FBS (Invitrogen). LLC-PK-1 cell line was grown in M199 supplemented with 3% FBS. HEI-OC1 cells were cultured in antibiotic free DMEM with 10% FBS (GIBCO) and 5 µg/ml of gamma interferon (Sigma) at 33°C and 10% CO_2_ in air. All the rest of cell lines were maintained at 37°C in an atmosphere of 5% carbon dioxide. Cisplatin (CDDP) and ebselen were purchased from Sigma and pan-caspase inhibitor Z-Val-Ala-Asp(OMe)-fluoromethylketone (zVAD-fmk) from Tocris. IDN-5665 was synthesized by Laboratorios Salvat, S.A. LipofectamineTM 2000 (Invitrogen) was used according to the manufacturer’s instructions to transfect HeLa cells with the control siRNA (Rsi; Cell Signaling) and Apaf-1 siRNA (Dharmacon and Cell Signaling).

### Cell-based caspase activation assay

MEFS and HeLa cells were seeded in 6-well plate at a cellular density of 1×10^5^ cells/well and 2×10^5^ cells/well, respectively, while 3×10^5^ cells/well were grown for LLC-PK-1. Cells were pre-treated with SVT compounds for 1 h and then cisplatin (25 µM) was added. After 30 h cells were harvested and cytosolic extracts were obtained as described previously [13]. Total protein (50 µg) was mixed with assay buffer containing 20 µM Ac-DEVD-afc substrate. Activity was measured using a Victor 2 spectrofluorimeter. HeLa cells were treated in the same conditions after 24 h of siRNA transfection. LLC-PK-1 were also pre-treated with SVT compounds for 1 h and then subjected to hypoxia/hypercapnia (HH - 1% O_2_, 18% CO_2_) conditions for 24 h plus 24 h of re-oxygenation before cytosolic extracts were prepared.

HEI-OC1 cells were plated in sterile 96-well microtiter plates (BD) at cellular density of 4500 cells/well and treated with the SVT compounds and the caspase inhibitor zVAD-fmk in a dose response manner. Apoptosis was induced with cisplatin and cells were incubated for 24 h. Caspase 3 activity was determined by using Caspase-Glo 3/7 Assay Kit (Promega) according to the manufacturer’s instruction.

### Cell viability assays

Cell proliferation was measured using a 3-(4,5-dimethylthiazol-2-yl)-2,5-diphenyltetrazolium bromide (MTT) colorimetric assay. MEFS, LLC-PK-1 and HEI-OC1 cells were grown in 96-well plates at a cellular density of 1500 cells/well, 2500 cells/well and 4500 cells/well, respectively. After seeding, the cells were left to adhere to the plate overnight and MEFS and HEI-OC1 were treated with cisplatin plus SVT compounds for 24 h at 37°C. Four hours before the end of the treatment MTT (1 mg/ml in PBS) was added to each well and the plates were incubated for a further 4 h at 37°C. Finally, the medium was removed and the precipitated formazan crystals were dissolved in optical grade DMSO. Plates were read at 570 nm on a Wallac 1420 workstation. Same procedure was achieved in the case of LLC-PK-1 cells after 24 h of HH plus 24 h of re-oxygenation.

### Cytochrome c release assay

MEFS and HeLa cells were grown in 6-well plate at the same conditions described above. After 24 h in the presence of SVT compounds, mitochondrial Cyt*c* was followed using the InnocyteTM Flow Cytometric Cytochrome c Release kit (Calbiochem). Same procedure was applied for HEI-OC1 cells upon 48 h of CDDP administration in the presence or not of the SVT compounds or the caspase inhibitor zVAD-fmk. Cells were analysed on a Cytomics FC 500 (Beckman Coulter) flow cytometer.

### Cytochrome c release assays in purified mitochondria

Purified mitochondria were prepared from wtMEFS cells. Briefly, cells were mechanically broken using a 2 ml glass/glass dounce homogenizer (Kontes), using two rounds of 30 strokes. Homogenates were cleared in MB buffer (10 mM Hepes pH 7.5, 210 mM mannitol, 70 mM sucrose, and 1 mM EDTA) at 1,500 g and the mitochondria were spun down at 10,500 g. For Cyt*c* release assays, 20 mg of purified mitochondria were resuspended in RB buffer (125 mMKCl, 5 mM succinate and 0.5 mM EGTA) supplemented with protease inhibitor cocktail (Roche). Proteins at indicated concentrations were added to the samples and the incubations were carried out at 30°C. At the indicated time points, samples were spun down and supernatants and pellets were analyzed by immunoblotting using anti-cytochrome *c* or anti-Apaf-1 antibodies.

### Clonogenicity assays

1250 MEFS cells per well were seeded in 24-well plates, treated with cisplatin 5 µM in the presence or absence of SVT016426. After 6 h of treatment, the apoptotic stimulus was removed. Two weeks latter cell were fixed with 2% paraformaldehyde and stained with crystal violet.

### Immunoblotting

Whole cell extracts were obtained by lysing cells in 25 mMTris-HCl pH 7.4, 1 mM EDTA, 1 mM EGTA, and 1% SDS plus protease and phosphatase inhibitors. Protein concentration was determined by the BCA protein assay. Lysates were subjected to SDS-PAGE, transferred to nitrocellulose membranes, and immunoblotted following standard procedures. The antibodies used were Apaf-1 (611364 from BD), cytochrome *c* (556433 from BD), capase-9 (9508 from Cell Signaling) and α-tubulin (T-8203 from Sigma). Quantifications were performed with Image J software.

### RT-qPCR

Total RNA was isolated using the RNAsy Kit (Qiagen) according to the manufacturer’s instructions. First strand cDNA was synthesized from 500 ng of RNA using Superscript First Strand Synthesis System (Invitrogen). Gene products were analyzed by qPCR using SYBR Green Mix (Roche) and specific oligonucleotides in a Roche 480 Lightcycler. Primer sequences are the following: F 5′-CTGGCAACGGGAGATGACAATGG-3′ and R 5′-AGCGGAGCACACAAATGAAGAAGC-3′ for Apaf-1 and F 5′-CTGGACGGAGTAGCTCCAAG-3′ and R 5′-GACGCTCGTGTTCCTCATGC-3′ for Bnip3. Each value was normalized to β-actin (F 5′-GGACTTCGAGCAAGAGATGG-3′ and R 5′-AGCACTGTGTTGGCGTACAG-3′) and expressed as relative units (R.U.). All reactions were performed in triplicate.

### Zebra fish

Zebrafish (Daniorerio) embryos of the AB wild-type strain were used. At 5 days post-fertilization (dpf) larvae were dispensed in 24 well plates in embryo medium in a tissue incubator at 28.5°C. Cisplatin at the concentration of 10 µM and compounds were added and incubated for 24 h. After this period of treatment, the embryos were stained with 0.01% DASPEI (Molecular Probes), washing and anesthetized with 0.04% of tricaine. In order to take photomicrographs, the embryos were mounted in methylcellulose for observation under a fluorescence microscope. Pictures obtained were analyzed by counting the number of neuroblast presented in the lateral line of embryos.

### Renal hot ischemia assay

This study was approved by the Committee on the Ethics and Animal Welfare of the CIPF (Permit Number: 10-0185). All efforts were made to minimize suffering.

Male Wistar rats (200 g) were distributed in 4 different groups (n = 5); vehicle, ischemia plus vehicle; ischemia plus 10 or 20 mg kg^−1^ SVT016448. Rats were anesthetised (Pentobarbital 6 mg ml^−1^), sacrificed by cervical dislocation and maintained during 60 min at 37°C. Then kidneys were extracted by abdominal incision and divided into three parts. The first slice was frozen in liquid nitrogen, the second was fixed in formaldehyde 4% and the third was fixed in OCT. In the intraperitoneal assay (vehicle, methyl cellulose 0.5%, Tween80 0.1% in PBS) the treatments were administered 60 min before the rats were sacrificed. For the intravenous assay (vehicle, 30% of DMSO in PBS) compound treatments were administered 6 h before the sacrifice and the ischemia was prolonged by additional 90 min.

### Pathological analysis

Kidneys were analyzed in the SIAL (Integral services for Laboratory animals Barcelona, Spain). Briefly, kidneys fixed in formaldehyde were included in paraffin and slices stained with haematoxylin-eosin. Five levels of damage were established between no damage and serious lesions and scored from 1 to 5. Score 5 corresponds to severe lesions and Score 4 corresponds to moderate damage.

### Immunohistochemistry

Kidney slices (8 µm) were fixed in cold acetone and blocked with 5% goat serum, 0.3% Triton X-100 in PBS, incubated with 10 µl of caspase-3 active antibody (PE active caspase-3, BD Pharmigen) and mounted in Mowiol with DAPI. Confocal images of four different fields per sample were captured with a Leica microscope. Quantification of caspase-3 activity was performed evaluating the mean grey value of the different fields using the ImageJ software analysis.

## Supporting Information

File S1 This file contains supporting information for the Materials and Methods section, Figures S1–S8 and Table S1.
(DOCX)
