# Supplementary material for: Apaf-1 Inhibitors Protect from Unwanted Cell Death in In Vivo Models of Kidney Ischemia and Chemotherapy Induced Ototoxicity
Source: PLoS One. 2014 Oct 20;9(10):e110979. doi: 10.1371/journal.pone.0110979 (PMC4203855; doi:10.1371/journal.pone.0110979)
Supplement: File S1 — This file contains supporting information for the Materials and Methods section, Figures S1–S8 and Table S1. (DOCX) [file pone.0110979.s001.docx]

**Supporting Information**

**Apaf-1 inhibitors protect from unwanted cell death in *in vivo* models of kidney ischemia and chemotherapy induced ototoxicity**

Mar Orzáez*, Mónica Sancho, Sandra Marchán, Laura Mondragón, Rebeca Montava, Juan G. Valero, Olatz Landeta, Gorka Basañez, Rodrigo J. Carbajo, Antonio Pineda-Lucena, Jordi Bujons, Alejandra Moure, Angel Messeguer, Carmen Lagunas, Carmen Herrero and Enrique Pérez-Payá.

*To whom correspondence should be addressed. E-mail: [morzaez@cipf.es](mailto:morzaez@cipf.es)

**This PDF file includes**

Supporting Materials and Methods

Supporting Figures S1 – S8

Supporting Table S1

Supporting References

**Supporting Materials and Methods**

**Recombinant proteins**

Expression and purification of His-tagged Apaf-1 1-591, caspase-3 and caspase-9 was performed as reported previously (Riedl et al., 2005; Stennicke et al., 1999). Recombinant full length Apaf-1 (rApaf-1) was obtained using the baculovirus expression system as described previously (Malet et al., 2006).

**Caspase 9 activation assay**

Caspase 9 was activated with kosmotropic salts, in particular with Na-citrate as reported previously (Boatright et al., 2003). Briefly, 5 µM caspase-9 was pre-activated for 20 min in SC buffer (50 mM Na_2_HPO4, 150 mM NaCl, 1.5% sucrose, 0.05% CHAPS, 10 mM DTT, 0.7 M Na-citrate, pH 7.4) at RT. Once pre-activated, the complete reaction was diluted to 100 nM caspase-9 in assay buffer without Na-Citrate and incubated 20 min at RT in the presence or absence of SVT compounds. Enzyme activity was monitored by adding the Ac-LEHD-afc substrate (40 µM).

**Caspase 3 activation assay**

Apaf-1 inhibitors were incubated with caspase 3 (2 nM) in reaction buffer (100 mM NaCl, 50 mM Hepes pH 7.4, 10 mM DTT, 1 mM EDTA, 10% glycerol, 0.1% CHAPS) for 20 min at RT. Enzyme activity was spectrophotometrically monitored (λ = 570 nm) by adding Ac-DEVD-pNA (200 µM) substrate.

**β- lactamase assay**

SVT compounds were tested for inhibition of β-lactamase. Recombinant protein was expressed and purified as described (Weston et al., 1998). Inhibitors and 1 nM enzyme were incubated for 20 min in 50 mM potassium phosphate buffer, pH 7.0, at RT. Following compound incubation, 200 μM CENTA (CalBiochem - β-lactamase substrate) was added to each sample and the absorbance was monitored at 405 nm to determine the activity.

**Cell cycle assay**

HDFn primary cells were maintained for nine passage and the cells were passed twice a week to avoid that the cells become over confluent. At the indicated passage, part of the culture were harvested and fixed in 70% ethanol at 4ºC. Then were stained in Tris-buffered saline containing propidium iodide (50 mg/ml), RNase A (10 mg/ml) for 1 h at 4ºC and analyzed on a FACS CANTO II flow cytometer (Becton-Dickinson). Data was analyzed by ModFit LF version 3.2 Software.

**Cell growth curves**

HDFn primary cells were seeded at 5000 cell/well in 24-well culture plates. Cells were treated or not with compounds (10 µM). Quadruplicate wells of each treatment were counted at indicated days until the seventh day after seeding. Cell counting was performed with a Neubauer chamber using trypan blue vital staining.

**
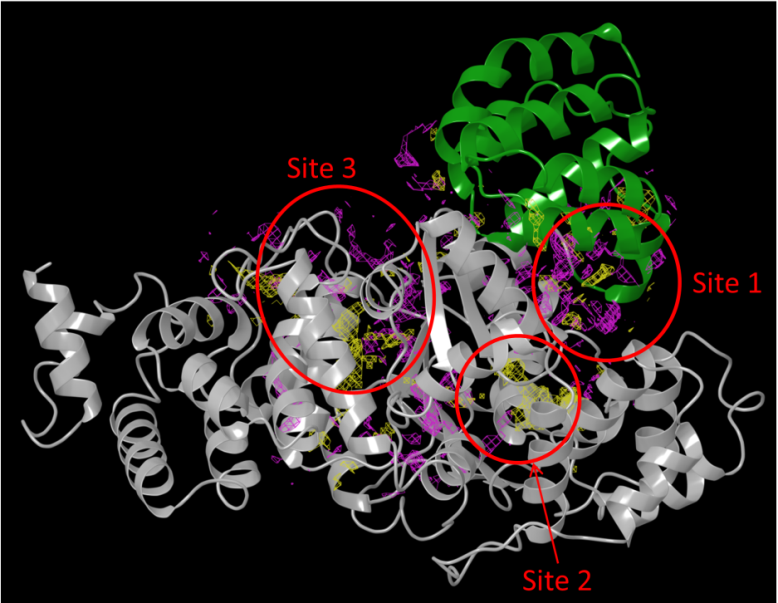
**

**Figure S1.** Apaf-1 1-591 structure showing the two domains (CARD in green, NOD in gray) and the computationally identified ligand binding sites, mapped as hydrophilic (magenta) and hydrophobic (yellow) mesh surfaces. Three main potential sites were identified. Site 1, located on a large cleft at the CARD-NOD interface and formed by helices α2, α3, α5, α7, α8, and the loop between residues 117-129. Site 2, coinciding with the ADP binding site, which implies helices α10, α15 and α17, sheets β2, β6 and β7, and loops between residues 117-129, 154-159 and 389-394. Interestingly, in the crystal structure the first and the second binding sites are connected through a narrow channel, suggesting that access to the deeply buried nucleotide binding site requires unpacking of the CARD-NOD interface. Site 3, located at a large cavity delimited by helices α12 and α25, sheets β2, β6 and β7, and loops between residues 207-219, 386-397 and 468-479. This hypothetical site, did not match with experimental data obtained by NMR and fluorescence polarization spectroscopy, therefore it was not considered further.

**
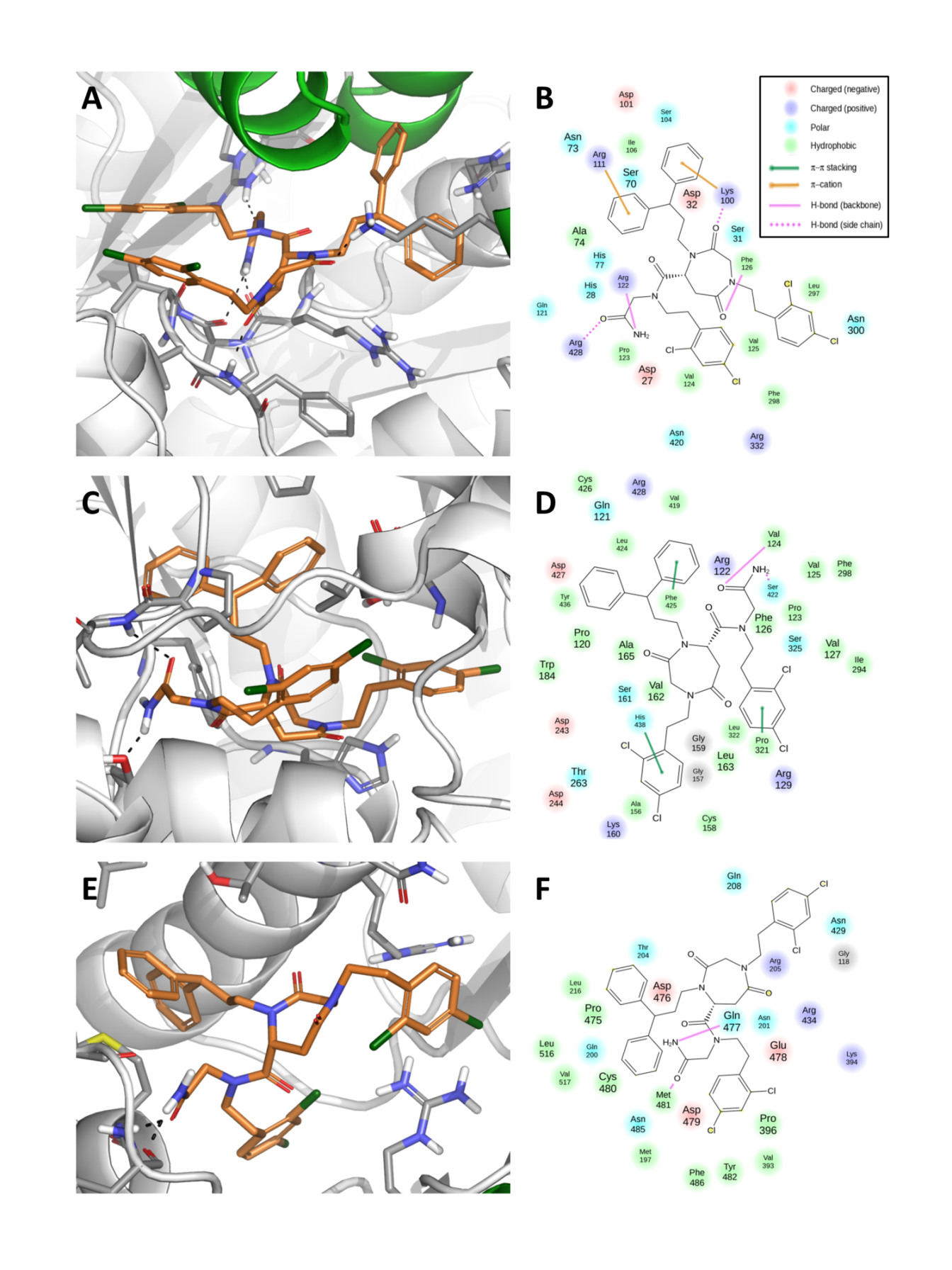
**

**Figure S2.** Best docked poses (**A** and **C**) and interaction diagrams (**B** and **D**) obtained for SVT016426 bound to Sites 1 (**A** and **B**) and 2 (**C** and **D**). The interaction diagrams show the Apaf-1 residues with atoms which are at less than 4 Å from the ligand.

**Figure S3.**  Dose response curves of Apaf-1 inhibitors analyzed using *in vitro* reconstitution of apoptosome (**A**) and HEK293 cell extract-based (**B**) assays. Curve fitting was performed using GraphPad Prism 3.0 software. Data are presented as mean inhibition percentage of control ± SD, n=3. (**C**) Activation of procaspase-9 by a forced dimerization-based assay is not inhibited by Apaf-1 inhibitors. Procaspase-9 processing is activated by dimerization in Na-citrate buffer in the presence of 10 µM of each Apaf-1 inhibitor. LEHDase activity was measured in a Wallac-Victor spectrofluorimeter workstation. (**D**) Caspase-3 *in vitro* activity is not affected by Apaf-1 inhibitors. Recombinant caspase-3 was incubated in the presence of 10 µM of each Apaf-1 inhibitor and DEVDase activity was followed in a Wallac-Victor spectrofluorimeter workstation**.** (**E**) Apaf-1 inhibitors do not modify β-lactamase activity. Activity in the presence of SVT compounds was measured by hydrolysis of CENTA substrate. CTRL+ means the activity of the well described β-lactamase aggregation-dependent inhibitor miconazol (50 μM)(Feng et al., 2007). All experiments were performed by triplicate. Data is presented as mean ± SD.


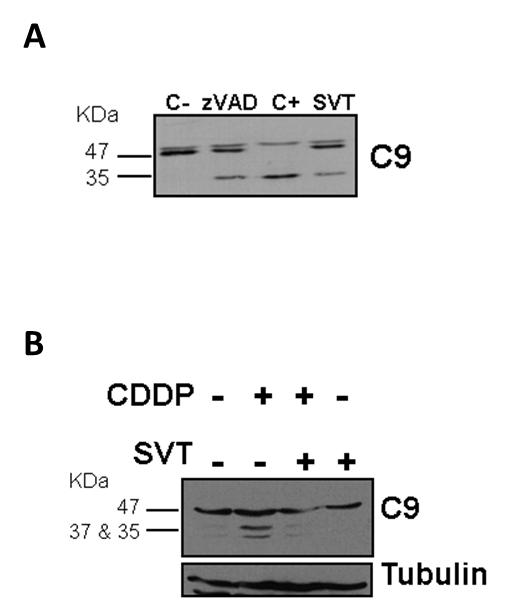


**Figure S4.** Apaf-1 inhibitor, SVT016426, decreases caspase-9 (C9) processing. (A) Immunoblot of C9 in the HEK293 cell extract-based assay described in Material and Methods. C- refers to cell extract depleted from Apaf-1. C+ means cell extract (depleted from Apaf-1) reconstituted with rApaf-1. zVAD and SVT correspond to samples in the same conditions as C+ but incubated with zVAD (1 μM) and SVT016426 (10 μM). (B) Immnunoblot of C9 in HeLa cells after incubation with CDDP (20 µM) in the presence of vehicle (DMSO) or SVT016426 (SVT; 10 µM). Tubulin is used as loading control.

**Figure S5.** Apaf-1 is required for the inhibitory activity of other Apaf-1 inhibitor-derivatives. (**A**) From left to right, cell survival, caspase-3 activity, and mitochondrial Cyt*c* of HeLa cells after incubation with CDDP (20 µM) in the presence of vehicle (DMSO), zVAD (1 µM), IDN6566 (IDN; 1 µM), Ebselen (10 µM), or SVT016426 (SVT; 10 µM) (mean ± SD, n = 3) (**B**) Apaf-1 was silenced (80% as determined by quantitative western blot) in HeLa cells upon two different Apaf-1 siRNA (100 nM) transfection. Bar graph represents the ratio between Apaf-1 band and tubulin in the western blot. (**C**) Caspase-3 activity and cell viability in HeLa cells transfected with control (Rsi) or Apaf-1 (Apafsi) silencer for 24h and treated with Apaf-1 inhibitor-derivatives. Cells were analyzed after 30 h incubation with cisplatin (CDDP; 20 µM) in the presence of vehicle (DMSO), SVT1016448 (10 µM), SVT017686 (10 µM) and SVT017923 (10 µM). The values represent the mean ± SD of three experiments. In all cases asterisks indicate significant differences (*** p* < 0.05) as determined by one way ANOVA test with Bonferroni’s multiple comparison post-test.

**Figure S6.** SVT016426 recovers mitochondrial membrane potential. Membrane potential study through DIOC_2_(3) staining in HeLa cells upon CDDP treatment (20 µM) and SVT016426 (10 µM).

**Figure S7.** SVT016448 recovers damage caused by renal hot ischemia. (**A**) Intravenously injected SVT016448 (10 mg/Kg) decreases caspase activity in a renal hot ischemia *in vivo* model. A representative image of immunofluorescence of active caspase-3 (left panel) and quantification (right panel). (**B**) Damaged tissue decreases in the presence of Apaf-1 inhibitor as determined by histopathological evaluation (see Supporting Information). (**C**) SVT016448 treatment-dependent decrease in Apaf-1 mRNA levels as analyzed by RT-qPCR. Data represent the mean ± SD, n ≥ 3. Asterisk indicates significant differences (** p* < 0.05) relative to ischemia control versus treatment as determined by *t*-student test.

**Figure S8.** Treatment with Apaf-1 inhibitors for long time does not cause proliferation alterations. (**A**) Effect of Apaf-1 inhibitors on proliferation of primary fibroblast (HDFn). HDFn cells were seeded in 24-well dishes and treated or not with the compounds (10 µM). Cell growth was monitored by cell counting over the 7 following days. Results are the mean ± SEM of four separate samples. (**B**) Incubation with Apaf-1 inhibitors does not induce aneuploidy in HDFn cells. At the indicate passage number (p5, p7 and p9), cells treated or not with the compounds (10 µM) were collected, permeabilized, stained with PI and DNA content measured by flow cytometry. The percentage of cells in each cell cycle phase was determined using ModFit LT software.

**Table S1.** Site scores for the best sites identified and docking scores for the best poses obtained for compound SVT016426. Residues that stabilize the docked poses through hydrogen-bonding, hydrophobic, π-cation or π-stacking interactions are listed.

|  | Site score^a^ | Docking score^b^ | H-Bond | Hydrophobic | π-cation | π-stacking |
| --- | --- | --- | --- | --- | --- | --- |
| Site 1 | 1.07 | -6.86 | Lys100 Arg122 Phe126 Arg428 | Ile106, Val124 Val125, Leu297 | Lys100 Arg111 |  |
| Site 2 | 1.14 | -8.52 | Val124 Ser422 | Pro120, Pro123 Val124, Val127 Val 162, Ala165 Trp184, Ile294 Pro321, Leu322 Val419 |  | Phe425 His438 |

^a^ SiteMap scores ≥ 1.0 suggest sites with properties (hydrophobicity, hydrophilicity, H-bond donor or acceptor, and metal binding properties) of high suitability for ligand binding. ^b^ Glide XP docking scores in kcal mol^-1^.

**Supporting References**

**Boatright, K.M., M. Renatus, F.L. Scott, et al. (2003). A unified model for apical caspase activation. Mol Cell. *11*, 529-541.**

**Feng, B.Y., A. Simeonov, A. Jadhav, et al. (2007). A high-throughput screen for aggregation-based inhibition in a large compound library. J Med Chem. *50*, 2385-2390.**

**Malet, G., A.G. Martin, M. Orzaez, et al. (2006). Small molecule inhibitors of Apaf-1-related caspase- 3/-9 activation that control mitochondrial-dependent apoptosis. Cell Death Differ. *13*, 1523-1532.**

**Riedl, S.J., W. Li, Y. Chao, et al. (2005). Structure of the apoptotic protease-activating factor 1 bound to ADP. Nature. *434*, 926-933.**

**Stennicke, H.R., Q.L. Deveraux, E.W. Humke, et al. (1999). Caspase-9 can be activated without proteolytic processing. J Biol Chem. *274*, 8359-8362.**

**Weston, G.S., J. Blazquez, F. Baquero, et al. (1998). Structure-based enhancement of boronic acid-based inhibitors of AmpC beta-lactamase. J Med Chem. *41*, 4577-4586.**
